# A Pilot Proof-of-Concept Study to Evaluate the Physiological and Cognitive Impacts of *Hericium erinaceus* on Healthy Adults

**DOI:** 10.3390/nu18193177

**Published:** 2026-09-26

**Authors:** Cassandra Evans, Douglas Kalman, Jacqueline Goodrich, Victoria Burgess, Jaime L. Tartar, Lia Jiannine, Flavia Pereira, Anthony Ricci, Antonella Schwarz, Jose Rojas, Elizabeth Esposito, Antonio Crisanti, Hailey Fuentes, Drewann Fearon, Kendall Andries, Jose Antonio, Alexis Madelyn-Adjei

**Affiliations:** 1 Department of Exercise Science, Dr. Kiran C. Patel College of Osteopathic Medicine, Nova Southeastern University, Fort Lauderdale, FL 33328, USA; 2 Substantiation Sciences, LLC, Weston, FL 33332, USA; 3 Nutrition Department, Dr. Kiran C. Patel College of Osteopathic Medicine, Nova Southeastern University, Fort Lauderdale, FL 33328, USA; 4 Department of Psychology and Neuroscience, Nova Southeastern University, Fort Lauderdale, FL 33328, USA; 5 Department of Human and Sport Performance, Rocky Mountain University of Health Professions, Provo, UT 84606, USA; 6 Department of Exercise Science, Barry University, Miami, FL 33161, USA; 7 School of Medicine, Anglia Ruskin University, Chelmsford CM1 1SQ, UK

**Keywords:** cognitive function, lion’s mane, dietary supplements, nootropics, nutrition

## Abstract

**Background**: Executive function is a critical component of cognitive health that supports goal-directed behavior, attentional control, and cognitive flexibility. These processes are sensitive to aging and lifestyle factors and may be modifiable through nutritional strategies. Hericium Erinaceus (lion’s mane; LM) is an edible mushroom containing bioactive compounds shown to stimulate neurotrophins such as brain-derived neurotrophic factor (BDNF), suggesting potential cognitive benefits. **Methods**: This pilot, proof-of-concept study examined the effects of four weeks of daily LM supplementation on executive function and circulating plasma BDNF in healthy adults. Participants (*n* = 10) were healthy adults (18–55 years) and consumed 2 g/day of a certified organic LM supplement composed of mycelial biomass and fruiting body for 28 days. Executive function was assessed using the NIH Toolbox Flanker Inhibitory Control and Attention Test and the dimensional change card sort (DCCS) test. Plasma BDNF concentrations were measured using a quantitative ELISA. Paired-samples *t*-tests were used to assess changes from pre to post, and effect sizes were calculated using Cohen’s d. **Results**: No statistically significant differences were observed in executive function or plasma BDNF following supplementation (*p* > 0.05). However, small-to-moderate effect sizes were observed for flanker inhibitory control and attention (Cohen’s d = 0.48), DCCS (Cohen’s d = 0.25) and plasma BDNF (Cohen’s d = 0.31), suggesting modest improvements from baseline. **Conclusions**: The results of this small, pilot, proof-of-concept study demonstrate, within the confines of this study, that LM is safe and well tolerated in healthy adults. The small-to-moderate effects on cognitive function combined with the modest increase in plasma BDNF suggest a promising signal for LM supplementation that warrants further investigation in larger and longer-term randomized double-blind placebo-controlled studies.

## 1. Introduction

Cognitive function encompasses the mental processes that allow individuals to acquire, process, store, and apply information. These processes are essential for performing everyday tasks and adapting to environmental demands, supporting daily activities, occupational performance, and independent living. Cognitive function is not static and can change with age, as well as lifestyle-related factors [1]. Executive function represents a subset of higher-order cognitive processes, including inhibitory control, working memory, and cognitive flexibility, that govern goal-directed behavior, attentional regulation, and adaptive responding. Executive functions rely on the integrity of distributed neural networks involving the prefrontal cortex, anterior cingulate cortex, and related brain regions [2]. Inhibitory control enables the suppression of irrelevant or distracting information. Cognitive flexibility, also referred to as task switching or set shifting, supports the ability to adapt thoughts and behaviors in response to changing task demands. These executive processes are essential for selectively attending to relevant stimuli, maintaining information in mind, and flexibly solving problems in dynamic environments. They are measured using assessments such as the NIH (National Institute of Health) Toolbox Flanker Inhibitory Control and Attention Test and dimensional change card sort (DCCS) test [2].

Executive functions are sensitive to aging-related changes but are modifiable through lifestyle behaviors. Research suggests increasing physical activity and intake of certain nutrients allow individuals to enhance or mitigate age-related changes in cognitive function [3,4,5]. As interest in strategies to support cognitive health continues to grow, the use of dietary supplements to support cognitive health has increased. One such supplement is *Hericium Erinaceus* (*H. Erinaceus*), or lion’s mane mushroom (LM). *H. Erinaceus* (lion’s mane) is an edible mushroom native to North America, Europe, and Asia and has a long history of use in traditional Chinese medicine.

Bioactive compounds found *H. Erinaceus* are shown to stimulate the synthesis of neurotrophins, such as nerve growth factor (NGF) and brain-derived neurotrophic factor (BDNF), which support neuronal survival, neurite outgrowth, and synaptic function [6,7]. These effects are thought to be mediated in part through actions on astrocytes and broader neural signaling pathways. Animal models show *H. Erinaceus* increases the production of key proteins such as nerve NGF and BDNF. This is thought to occur in the hippocampus, a region that is critical for learning and memory [8]. BDNF supports the growth, survival, and communication of neurons, as well as synaptic plasticity, which underlies learning and memory formation [9]. Animals consuming *H. Erinaceus* demonstrate better learning ability and improved recognition memory, likely due to increased NGF and BDNF enhancing neural connectivity and efficiency [8,9].

Previous work has shown mood-enhancing effects following chronic administration of *H. Erinaceus*, with both studies showing improvements in depression and anxiety scores [6,10]. Of particular relevance, Vigna et al. [6] conducted a pilot clinical study examining eight weeks of *H. Erinaceus* supplementation in 77 adults with overweight or obesity who presented with mood and/or sleep disorders—a clinical population meaningfully different from the healthy adults enrolled in the present study. That study reported significant reductions in depression, anxiety, and sleep disorder scores and, notably, measured circulating pro-BDNF and BDNF using isoform-specific enzyme-linked immunosorbent assays (ELISAs). Vigna et al. observed a significant increase in serum pro-BDNF without a corresponding change in mature BDNF, raising important questions about proteolytic cleavage regulation and the functional significance of the pro-BDNF/BDNF ratio as a clinical biomarker. Critically, while that pilot study assessed neurotrophin isoforms in the context of mood outcomes, it did not evaluate cognitive performance directly. The present study extends this line of investigation by examining both circulating BDNF and objective measures of executive function in a healthy adult cohort, thereby addressing a gap left by prior work and directly linking neurotrophin signaling to cognitive performance outcomes. More recently, a study on the acute and chronic effect of *H. Erinaceus* suggested that it may improve speed of performance and reduce subjective stress in healthy, young adults [11]. Similarly, another recent study observed improvements in cognitive function and changes in circulating brain-derived neurotrophic factor (BDNF) levels, supporting the proposed neurotropic and neuroprotective effects of the bioactive metabolites present in *H. Erinaceus* [12]. When looking specifically at the impact of *H. Erinaceus* on memory, another study showed that oral intake of *H. Erinaceus* significantly improved cognitive function and prevented the deterioration of memory over time in older adults with cognitive decline [13]. Given the previous work in the literature on older adults and adults with co-morbidities, there is a need to understand the benefits of *H. Erinaceus* on cognitive health in a healthy adult population. In addition, given the variety of products on the market and the unique fingerprint of bioactive molecules in the mycelium and the fruiting body of *H. Erinaceus*, a study treating both the mycelium and the fruiting body would aid the understanding of this popular nutraceutical ingredient.

In addition to its neuroactive phytochemicals, *H. Erinaceus* contains B-vitamins, β-glucans, terpenoids, diterpenoids, polyphenols, sterols and essential minerals that support cognitive function [7]. Research demonstrates improvements in mood and cognitive function following supplementation with LM in doses ranging from 1 to 3 g/day [7,11,13,14,15]. One study observed changes in BDNF suggesting enhanced activation of BDNF-related pathways involved in synaptic plasticity [6]. These findings suggest that LM may represent a promising nutritional strategy for supporting executive function through neurotrophin-related mechanisms; however, controlled human studies evaluating its effects on both cognitive performance and circulating BDNF remain limited. Importantly, prior studies that measured BDNF isoforms—such as that of Vigna et al. [6], which specifically tracked pro-BDNF and the pro-BDNF/BDNF ratio as potential clinical biomarkers—focused primarily on mood outcomes in overweight and obese individuals and did not include objective cognitive performance testing. The present study is distinct in that it directly measures circulating plasma BDNF using a quantitative sandwich ELISA alongside validated measures of executive function, enabling an examination of the potential relationship between neurotrophin signaling and cognitive outcomes in a healthy adult population.

*H. Erinaceus* is a popular culinary mushroom species that is wild-harvested and cultivated in many countries. Thus, preparations made from liquid fermentation or solid-state fermentation processes provide a means to highly efficient, consistent, year-round production and allow for better quality control of the final product [16,17]. The current pilot, proof-of-concept study examined whether four weeks of daily lion’s mane mycelium biomass and fruit body supplementation could influence circulating BDNF levels and cognitive performance in healthy adults. It was hypothesized that supplementation might lead to modest improvements in executive function and expected to be safe and well tolerated.

## 2. Materials and Methods

### 2.1. Study Design

In this pilot, proof-of-concept early-stage study, participants reported to the clinical site on two separate visits. Participants were instructed to abstain from exercise on testing day and arrive at the lab fasted (no food or water 4 h prior). Testing sessions consisted of two visits (pre-testing/baseline and post-testing) with 28 days between visits. During the first visit, demographics and anthropometrics were assessed. At every testing visit, participants completed the NIH Toolbox Flanker Inhibitory Control and Attention Test and blood samples were collected. All participants underwent an informed consent process in accordance with the Declaration of Helsinki and an approved IRB protocol approved by the BRANY institutional review board, IRB number # 255200.

### 2.2. Participants

All participants were healthy, non-smoking adults (18–55 y.o.) with BMI 19–34.9 and recruited from the local community by standard recruitment methods. Participants were excluded from the study if they did not meet the inclusion/exclusion criteria for health status, were allergic to the study product or had a history of mental health/psychiatric treatment or hospitalization in the 12 months prior to the study. Inclusion/exclusion criteria were assessed via participant reporting and research staff review/confirmation. Participants were instructed to maintain a stable lifestyle with no change in exercise or diet for the duration of the study. Participants were instructed to abstain from consuming any mushroom containing dietary supplements for the study period (i.e., mushroom dietary supplement products and mushroom-inclusive drinks); see Table 1.

### 2.3. Supplement

The *Hericium Erinaceus* preparation used in this study is a certified organic powdered *H. Erinaceus* (strain M2-102-10, US patent 2024/0164424 A1) [18] mycelial biomass and fruiting body cultured on organic whole oats (*Avena sativa*) in a controlled solid state fermentation environment and then dehydrated and milled by M2 Ingredients, Inc (Vista, CA, USA). The powder was encapsulated in vegetable cellulose capsules, and each dose consisted of 2 g of *H. Erinaceus* powder (3 capsules). Participants were instructed to take 3 capsules daily.

### 2.4. Flanker Inhibitory Control and Attention

The NIH Toolbox Flanker Inhibitory Control and Attention Test (FICA) is a validated measure of executive function that assesses inhibitory control and attentional processing. The flanker task was administered via a touch-screen interface and consisted of a brief practice block followed by two test blocks (fish and arrows). Task instructions were presented visually on the screen, and a short practice block was completed to ensure task comprehension prior to testing. Detailed testing procedures are described by Zelazo et al. [2].

During test trials, participants were instructed to indicate the direction of a central stimulus while inhibiting responses to flanking distractors. No performance feedback was provided during test trials. Task performance was scored according to standardized NIH Toolbox procedures, with higher scores indicating better inhibitory control and attentional performance.

### 2.5. Dimensional Change Card Sort

The dimensional change card sort (DCCS) is a validated measure of executive function that assesses cognitive flexibility and working memory. The DCCS test was administered via a touchscreen interface and consisted of three test blocks. Task instructions were presented visually; on the screen, a short practice block was completed to ensure task comprehension before testing started. Detailed testing procedures are described by Zelazo et al. [2].

During test trials, participants were instructed to sort stimuli according to a specified rule (e.g., color or shape) and flexibly switch between rules when task demands changed. No performance feedback was provided during test trials. Task performance was scored according to standardized NIH Toolbox procedures, yielding age-corrected standard scores, national percentile ranks, and fully corrected T scores, with higher scores indicating better cognitive flexibility and executive function.

The study design decreased the chance of developing practice effects, as the FICA and DCCS were spread out by ~28 days between the testing.

### 2.6. Serum BDNF

Blood samples were collected between 1200 and 1400, gently inverted eight to 10 times immediately and then placed in a centrifuge and spun for 12 min at 3000 revolutions per minute (RPM) to separate serum from blood cells. All samples were stored at −80 °C within the following four hours to avoid possible changes in BNDF level until analysis. BDNF concentrations were determined using a quantitative sandwich ELISA (Human BDNF ELISA Kit, Abcam #ab212166, Cambridge, MA, USA).

Duplicate measurements were carried out at controlled room temperature and processed by the same operator to ensure consistency. The average of both duplicate measurements was used.

### 2.7. Statistical Analysis

All statistical analyses (intent to treat) were conducted using IBM SPSS Statistics (version 29.0.1.1). Descriptive statistics were computed for all participant demographics. A two-tailed paired-samples *t*-test was conducted to examine differences in pre- and post-testing for all outcomes. Effect sizes were calculated as Cohen’s d for within-subject designs, with 95% confidence intervals reported. Statistical significance was set at *p* < 0.05. Data are presented as mean ± standard deviation (SD).

## 3. Results

### 3.1. Participants

Ten participants (five males, five females) completed the study (Table 1).

### 3.2. Cognitive Testing

No statistical differences between pre- and post-testing were observed on both Flanker Inhibitory Control and Attention test and dimensional change card sort (Table 2 and Table 3 & Figure 1, Figure 2, Figure 3, Figure 4, Figure 5 and Figure 6). However, a small-to-moderate effect size was observed in both cognitive measures, suggesting modest improvements from pre- to post-testing.

### 3.3. BDNF Testing

No statistical differences were reported in pre- and post-plasma BDNF measures. Based on the effect size, Cohen’s d demonstrated a small-to-moderate magnitude of change (Cohen’s d = 0.31) (Table 4 and Figure 7).

## 4. Discussion

This pilot (small sample size) proof-of-concept (PoC) study investigated the effects of chronic LM supplementation on cognitive performance and circulating BDNF in healthy adults over a one-month period. Although no statistically significant changes in cognitive outcomes were observed in the PoC study, small-to-moderate effect sizes suggest modest improvements in executive function following supplementation. The observed effect size findings in this study are of interest and considered potential signals worthy of follow-up. The flanker test yielded up to a 0.48 Cohen’s d; a d value near 0.5 means that the average person in the group with the higher score would score higher than approximately 69% of the people in a potential comparison group or the general population. The DCCS data demonstrated up to a 0.45 Cohen’s d (medium/moderate effect), indicating that the average person in the group with the higher score would score higher than 60% of a comparison group or the general population. For the serum BDNF, the Cohen’s d was 0.31, indicating a small effect, meaning the average person in the LM group would score higher than approximately 62% of the people in a comparison group of the general population. The totality of the multitude of observed effect sizes in this PoC study provides rationale and evidence which ties together a potential mechanism of action (BDNF effects) with the cognitive outcomes as measured in healthy subjects (FICA, DCCS).

Importantly, the study product (LM) was well tolerated, with no adverse effects reported or observed, supporting its safety in healthy adults. These findings indicate that while cognitive effects may be subtle in this population, LM may influence executive processes, warranting further investigation in a proper prospective randomized double-blind placebo-controlled study that is adequately powered for the outcomes of interest.

The present findings can be meaningfully compared and contrasted with the pilot study by Vigna et al. [6], which represents the most directly relevant prior clinical investigation of *H. Erinaceus* and neurotrophin biomarkers. Vigna et al. enrolled 77 adults with overweight or obesity (body mass index ≥ 25 kg/m^2^) presenting with mood and/or sleep disorders, randomizing them to eight weeks of *H. Erinaceus* supplementation or a control condition, both under a low-calorie diet regimen. In contrast, the present study enrolled healthy adults (body mass index, 19–34.9 kg/m^2^; mean, 25.7 ± 4.2) without mood disorders or obesity-related comorbidities, providing a fundamentally different clinical context. These population differences are critical: in the study by Vigna et al., baseline mood dysregulation and metabolic dysfunction may have created greater physiological “room for improvement” in neurotrophin signaling, potentially amplifying observable effects relative to a healthy cohort where neurotrophin levels are likely closer to optimal at baseline.

Regarding BDNF outcomes, the two studies also differ importantly in design. Vigna et al. measured both circulating pro-BDNF and mature BDNF using isoform-specific ELISAs and reported a significant increase in serum pro-BDNF without a corresponding change in mature BDNF following supplementation—interpreting the elevated pro-BDNF/BDNF ratio as a potential index of altered proteolytic processing rather than straightforward neurotrophin upregulation. In contrast, the present study measured total plasma BDNF via a quantitative sandwich ELISA (Abcam #ab212166) without separately quantifying pro-BDNF. Although the present study observed a modest, non-significant numerical increase in plasma BDNF (Cohen’s d = 0.31), the absence of isoform-specific measurement limits direct comparability with the findings of Vigna et al. Taken together, the pattern across both studies suggests that *H. Erinaceus* may modulate the neurotrophin axis through effects on pro-BDNF processing and the pro-BDNF/BDNF ratio rather than producing large increases in circulating mature BDNF. This mechanistic nuance has potentially important implications for cognitive function: while mature BDNF acting via TrkB receptors is classically associated with synaptic plasticity, long-term potentiation, and memory consolidation, the biological role of pro-BDNF and its receptor p75NTR in higher cognitive processes, including executive function, is less well understood and warrants dedicated investigation in future trials.

Previous studies examining acute LM supplementation have reported mixed findings, with some evidence of selective improvements in psychomotor performance but not executive function [19,20]. La Monica et al. [20] observed selective improvements in reaction time and processing speed measures, with effects most evident at 120 min post-ingestion of 1 g of LM. In contrast, executive function benefits appear more likely to occur following chronic supplementation, which is consistent with the proposed neurotrophic mechanisms of LM. Evidence from chronic supplementation studies suggests that LM-related cognitive benefits may be population- and context-specific [7,13,14,15]. In older adults, 12 weeks of LM supplementation (3.2 g/day) was associated with improvements in Mini-Mental State Examination (MMSE) scores, reflecting gains in attention and memory [13]. Similarly, Li et al. [15] observed MMSE improvements following a lower daily dose (1 g/day) administered over a longer duration (49 weeks). Taken together, these findings suggest that LM’s cognitive effects are modest, domain-specific, and dependent on factors such as age, baseline cognitive status, dose, and duration of supplementation.

Few studies examine the effects of chronic supplementation in younger, healthy populations [11,21]. Similar to our findings, Grozier et al. [21] did not observe improvements in cognitive function/executive function in healthy college-aged adults. Participants consumed 10 g/day of *H. Erinaceus* for 4 weeks. Interestingly, cognitive testing was conducted while participants were performing a demanding dual task. The lack of cognitive decline suggests LM may offer potential support during times of stress. In another randomized, double-blind, placebo-controlled pilot trial in healthy adults who consumed LM (1.8 g/day) for 28 days [11], compared to the placebo, acute dosing of LM was associated with improved performance on Stroop but poorer immediate word recall. However, chronic supplementation resulted in lower levels of subjective stress. Similar to the present study, results must be interpreted cautiously due to the small sample size and differences in cognitive tests used.

The present study observed a numerical increase in serum BDNF, with a small-to-moderate effect size (Cohen’s d = 0.31), suggesting a modest trend toward higher circulating BDNF following supplementation. Preclinical evidence indicates that lion’s mane may exert neurotrophic effects in the brain. BDNF is produced as a precursor (pro-BDNF) that can be cleaved into mature BDNF (mBDNF), with mBDNF generally associated with neuronal survival, synapse formation, long-term potentiation, and memory-related processes [7,11]. However, peripheral BDNF is influenced by multiple physiological factors (e.g., sex hormones, physical activity) and may not directly reflect central neurotrophic signaling [8]. This may contribute to the variability observed in the current study. Notably, prior clinical work suggests LM may modulate pro-BDNF and the pro-BDNF/BDNF ratio without necessarily producing significant changes in circulating BDNF, highlighting the importance of assessing multiple BDNF-related biomarkers in future trials [7,8]. Interestingly, female participants demonstrated the largest numerical increases in plasma BDNF (Figure 8 and Figure 9), warranting further investigation in adequately powered studies.

This observation is consistent with the findings of Vigna et al. [6], whose pilot study in overweight and obese adults with mood disorders similarly found that *H. Erinaceus* supplementation significantly increased circulating pro-BDNF without a statistically significant change in mature BDNF—a divergent pattern from what a straightforward neurotrophin-upregulation hypothesis would predict. Importantly, whereas the study by Vigna et al. linked pro-BDNF changes to mood outcomes but did not assess cognitive performance, the present study is the first, to our knowledge, to pair quantitative plasma BDNF measurement with objective executive function testing—specifically the NIH Toolbox Flanker Inhibitory Control and Attention Test and dimensional change card sort—following *H. Erinaceus* supplementation in healthy adults. The co-occurrence of modest BDNF signal (Cohen’s d = 0.31) alongside small-to-moderate improvements in executive function (Cohen’s d = 0.42–0.48 for flanker measures), while not permitting causal inference at this sample size, provides preliminary proof-of-concept support for the hypothesis that *H. Erinaceus*’s impact on cognitive function may be mediated, at least in part, through BDNF-related neuroplasticity pathways. This putative mechanism—still in the proof-of-concept stage—further reinforces the need for adequately powered, randomized, placebo-controlled trials in healthy adults that include comprehensive neurotrophin profiling (both pro-BDNF and mature BDNF) alongside validated cognitive endpoints.

The primary limitation of this study is the small sample size, which reduces the ability to pick up on subtle cognitive benefits. Additionally, the sample included a greater proportion of male than female participants, which may have influenced plasma BDNF outcomes given known sex-related differences in neurotrophin regulation. Future studies should employ larger, more robust sample sizes combined with longer supplementation periods. Moreover, future studies should include comprehensive neurotrophin profiling (including pro-BDNF and mBDNF) and consider using cognitive tests that have been applied in prior LM studies or that are better suited to younger, healthy populations in order to capture more subtle cognitive changes. Importantly, the present study and the Vigna et al. [6] pilot study collectively represent early, proof-of-concept evidence for neurotrophin modulation by *H. Erinaceus* in humans, albeit in distinct populations. Neither study was powered for definitive conclusions. However, taken together, the convergent signals across mood outcomes (Vigna et al.), executive function measures, and BDNF trends (present study) provide a compelling scientific rationale for a well-powered, randomized, double-blind, placebo-controlled trial enrolling healthy adults in a similar age and body mass index range to the present study. Such a trial should prospectively include isoform-specific neurotrophin measurement (pro-BDNF, mBDNF), standardized cognitive assessments spanning multiple domains, a supplementation period of at least 12 weeks, and sufficient sample size to detect effects of the small-to-moderate magnitude observed here. Recruiting a demographically similar healthy adult population—rather than shifting to clinical populations with mood disorders or obesity as studied by Vigna et al.—would allow direct confirmation of the cognitive-neurotrophin signal suggested by the present proof-of-concept data.

## 5. Conclusions

The results of this proof-of-concept study demonstrate that the LM studied is safe and well tolerated in healthy adults. The small-to-moderate effects on cognitive function combined with the modest increase in plasma BDNF suggest a promising signal for LM supplementation that warrants further investigation in larger and longer-term randomized double-blind placebo-controlled studies.

## Figures and Tables

**Figure 1:**
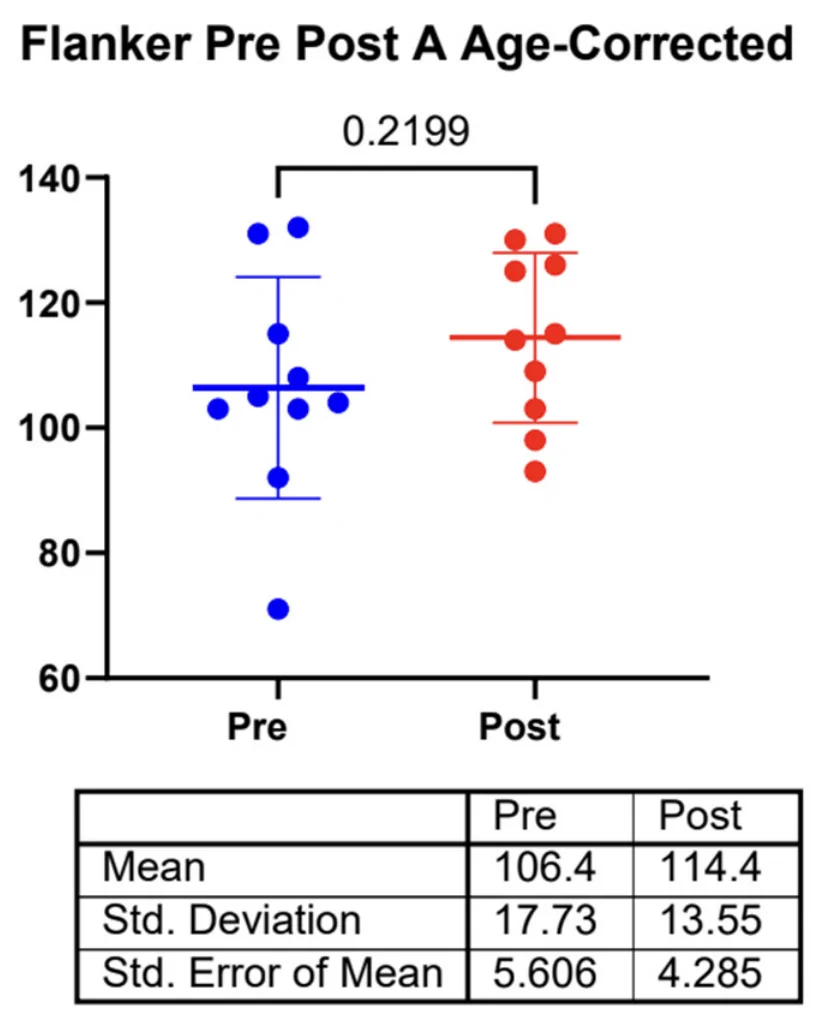
Flanker—individual data points for age-corrected standard score, Group A.

**Figure 2:**
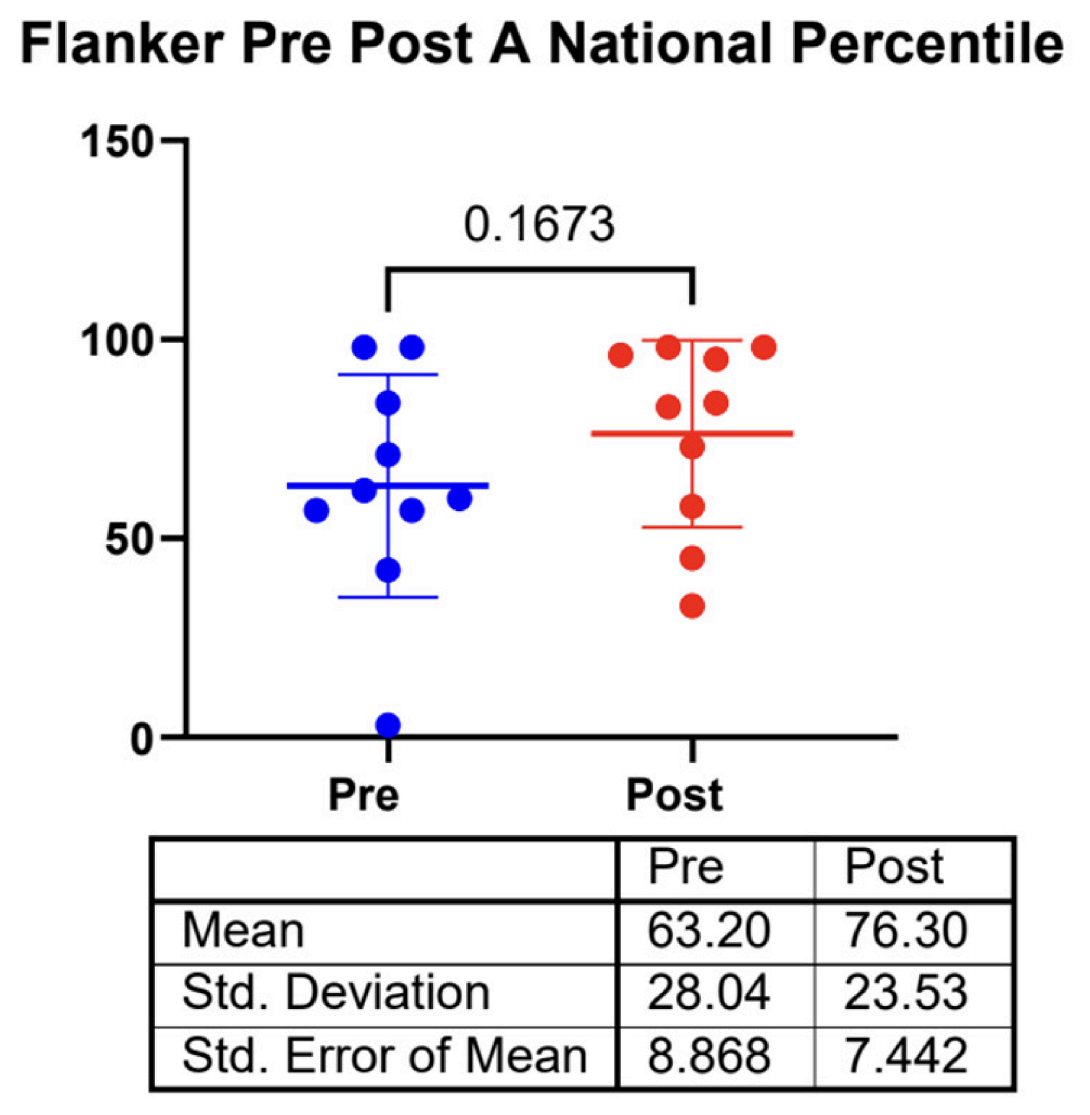
Flanker—individual data points for flanker national percentile (age-adjusted), Group A.

**Figure 3:**
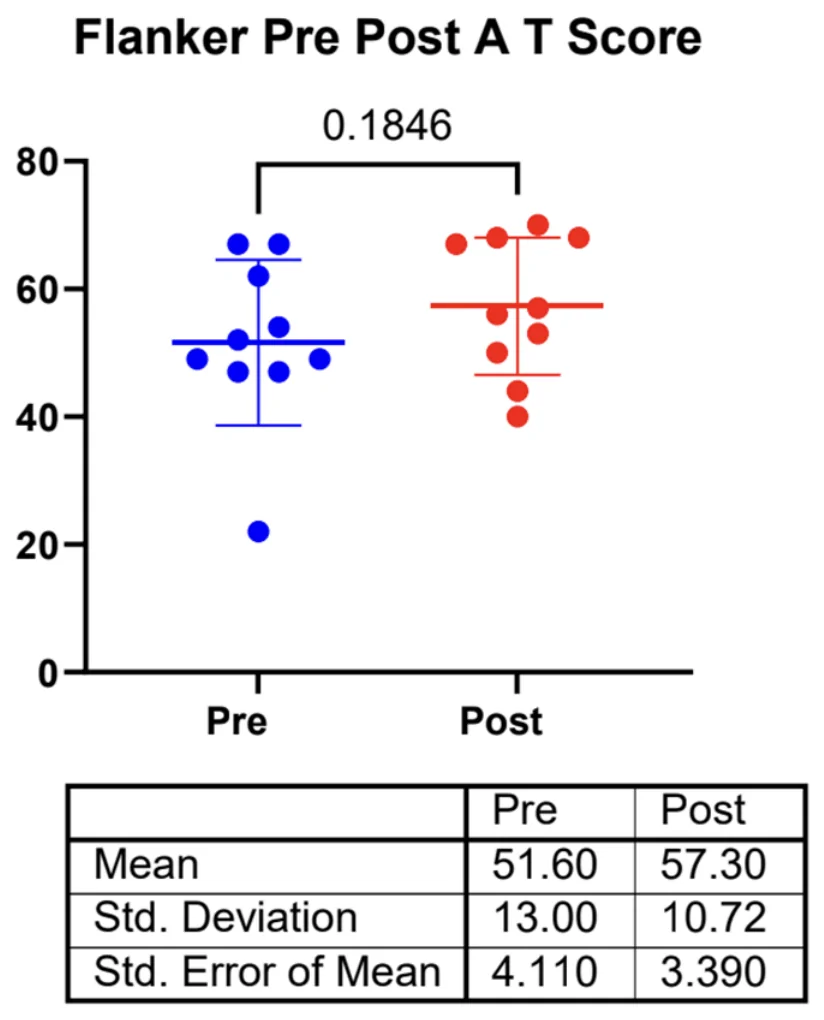
Flanker—individual data points for fully corrected T score, Group A.

**Figure 4:**
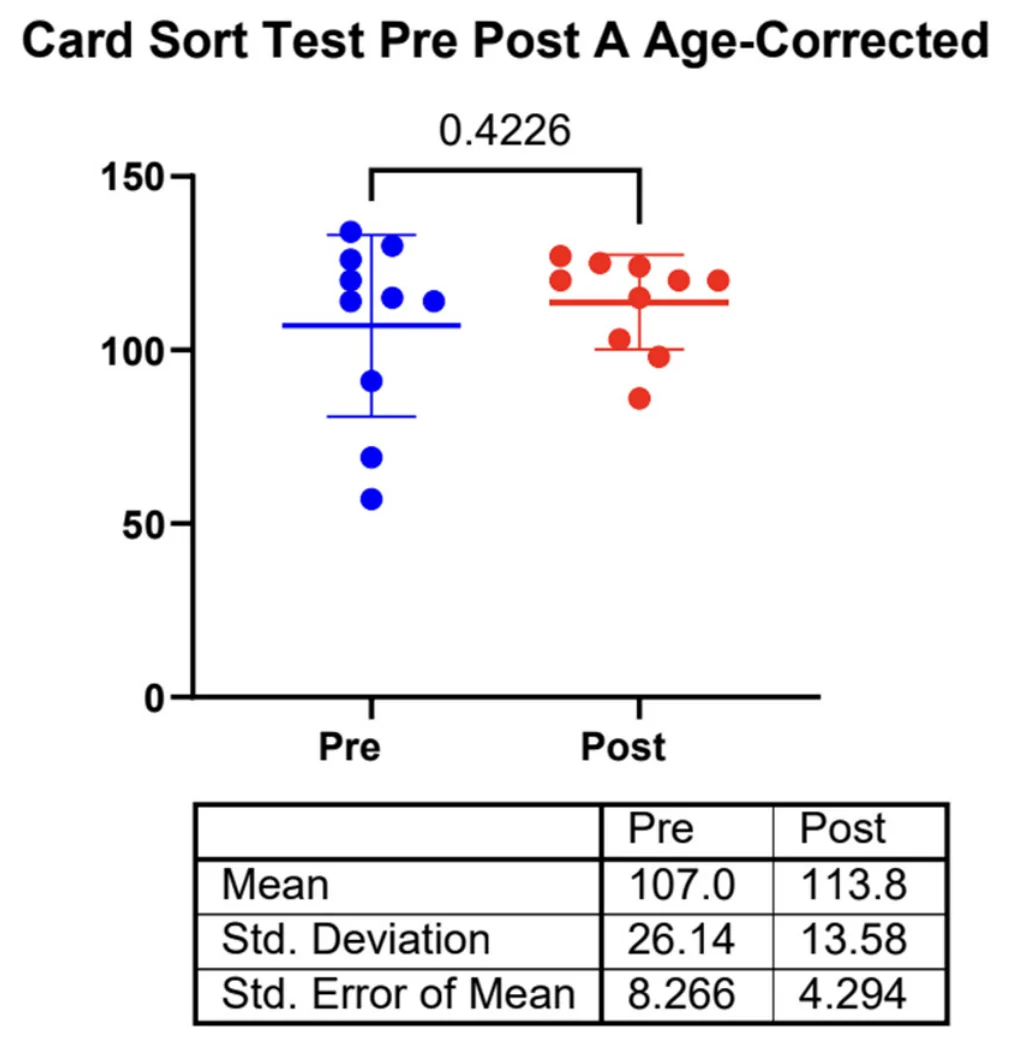
Dimensional change—individual data points for age-corrected standard score, Group A.

**Figure 5:**
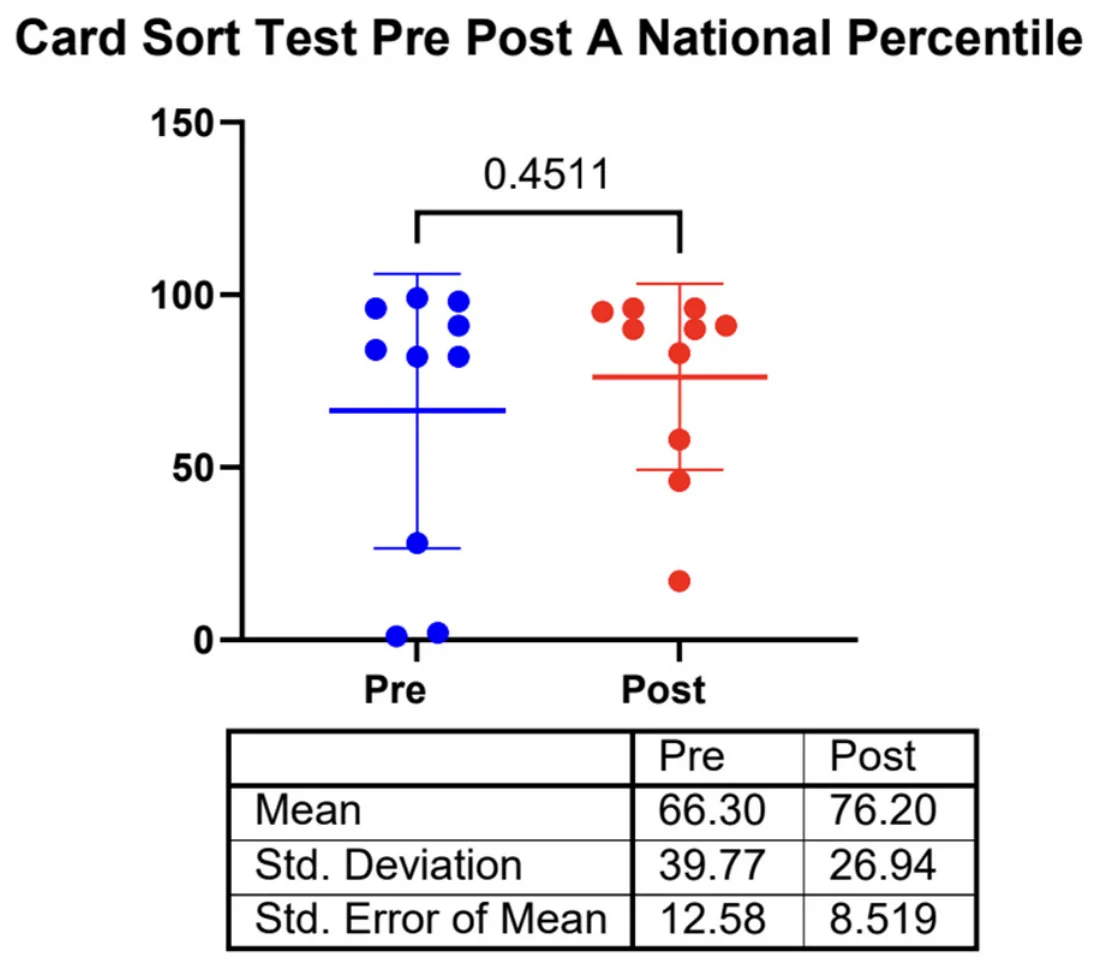
Dimensional change—individual data points for DCCS national percentile (age-adjusted), Group A.

**Figure 6:**
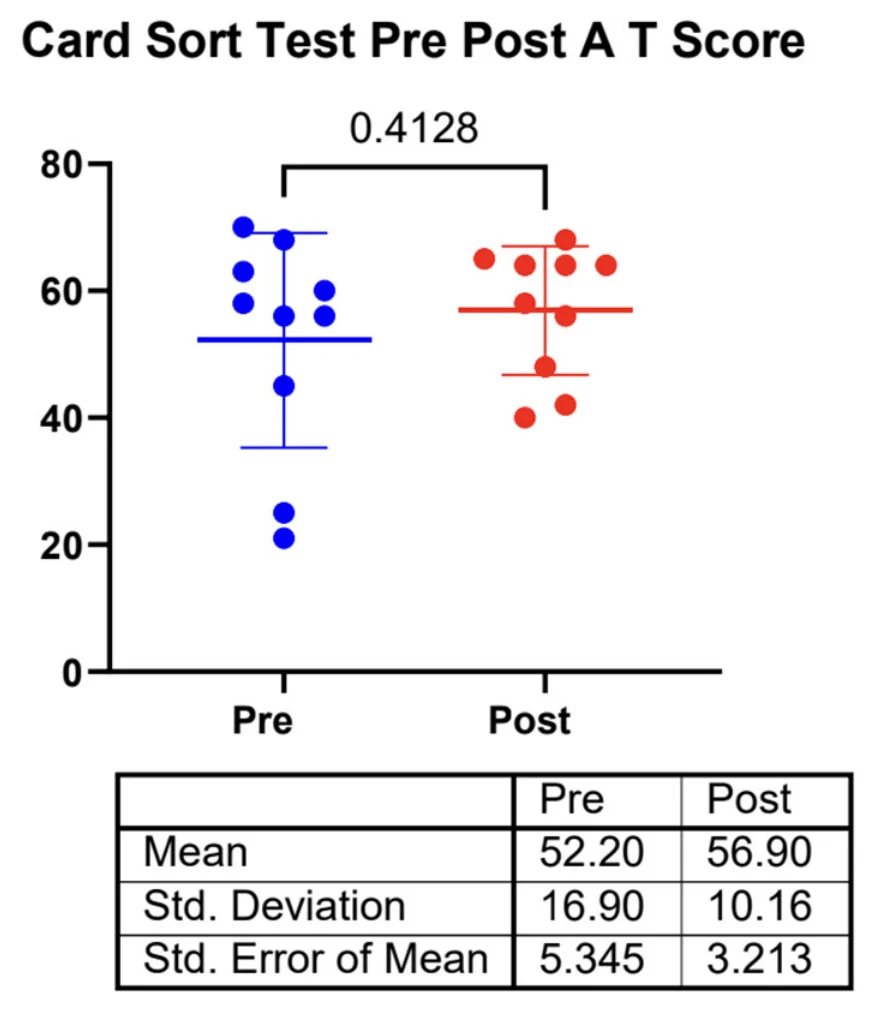
Dimensional change—individual data points for fully corrected T score, Group A.

**Figure 7:**
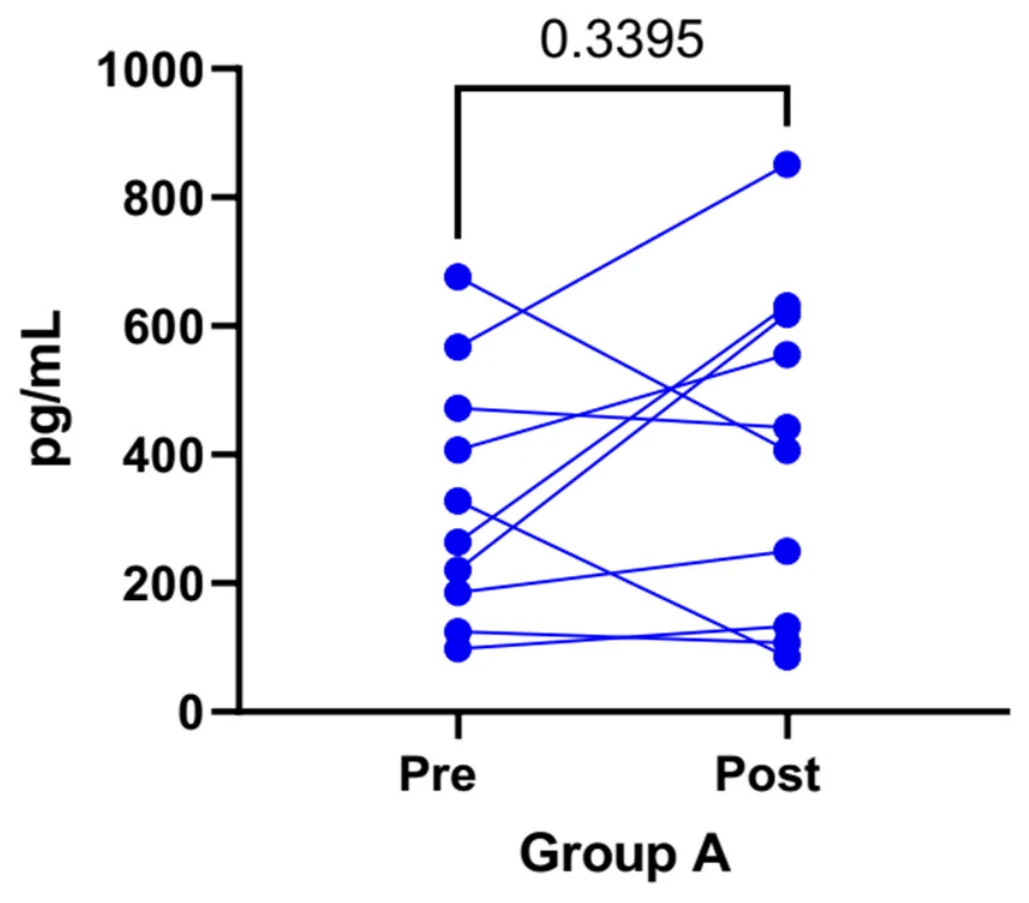
BDNF—Individual data points for Group A.

**Figure 8:**
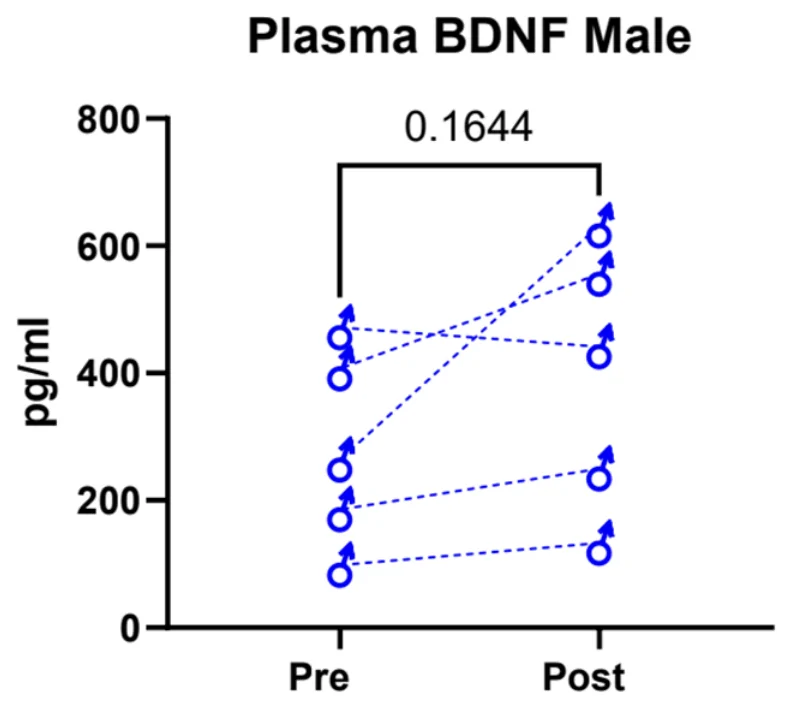
BDNF—pre- vs. post-treatment—male individual data points.

**Figure 9:**
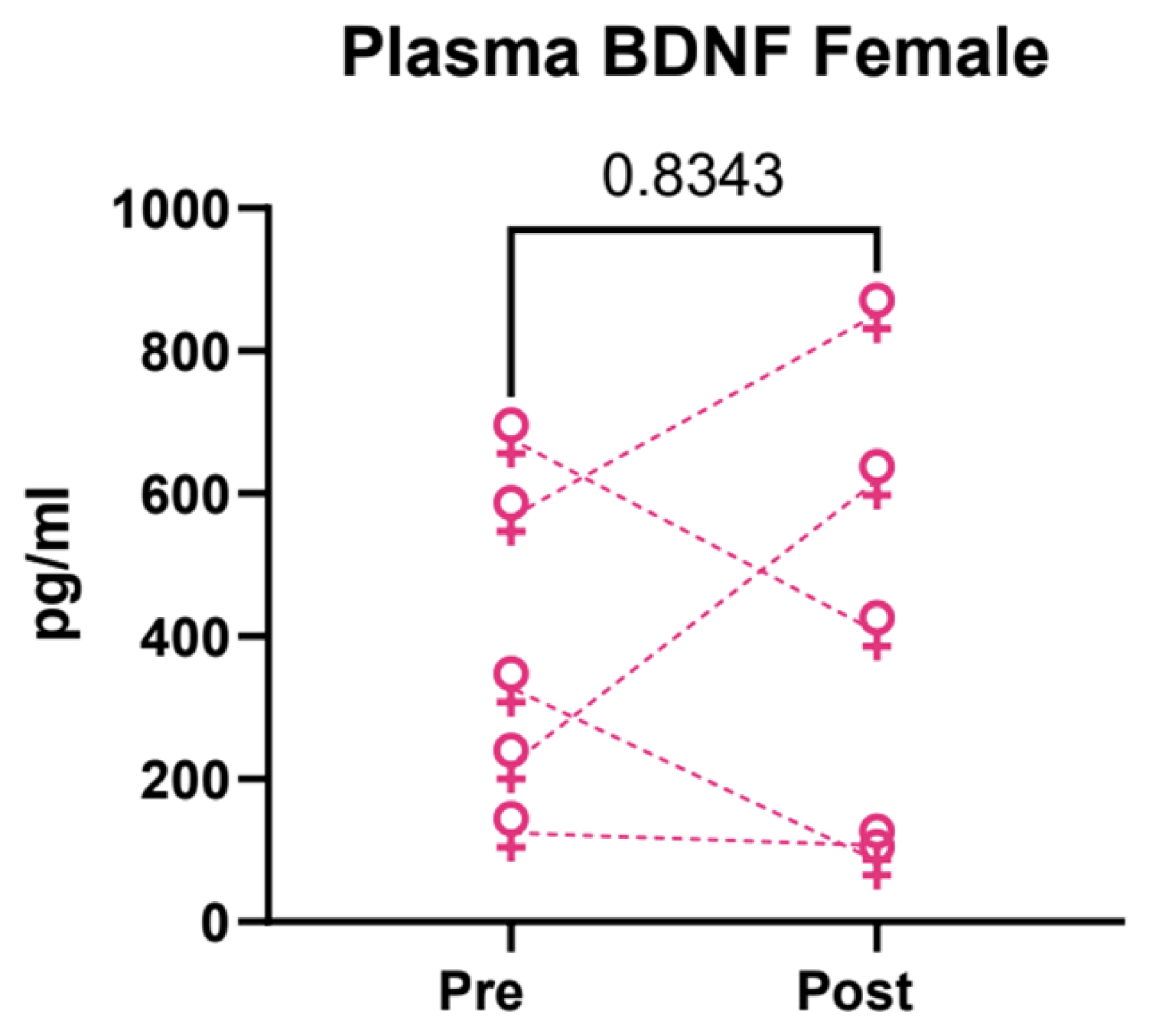
BDNF—pre- vs. post-treatment—female individual data points.

**Table 1:** Participant demographics ^1^.

Group	Age (Years)	Height (cm)	Weight (kg)	BMI
A (*n* = 10, 5 female)	33 ± 11	172.9 ± 12.4	77.3 ± 18.8	25.7 ± 4.2
B (*n* = 10, 6 female)	37 ± 11	169.3 ± 8.2	73.9 ± 20.7	25.5 ± 5.5

^1^ Data are expressed as the mean and standard deviation.

**Table 2:** Flanker Inhibitory Control and Attention Test.

	Pre (Mean ± SD)	Post (Mean ± SD)	*p*-Value	Cohen’s d	95% CI
Age-Corrected Standard Score	106.4 ± 17.7	114.4 ± 13.5	0.220	0.42	−0.22 to 1.29
Flanker National Percentile (Age-Adjusted)	63.2 ± 28.0	76.3 ± 23.5	0.169	0.48	−0.15 to 1.37
Fully Corrected T Score	51.6 ± 13.0	57.3 ± 10.7	0.184	0.45	−0.18 to 1.34

**Table 3:** Dimensional change card sort.

	Pre (Mean ± SD)	Post (Mean ± SD)	*p*-Value	Cohen’s d	95% CI
**Age-Corrected Standard Score**	107.0 ± 26.1	113.8 ± 13.6	0.423	0.27	−0.36 to 0.90
**DCCS National Percentile (Age-Adjusted)**	66.3 ± 39.8	76.2 ± 26.9	0.451	0.25	−0.38 to 0.88
**Fully Corrected T Score**	52.2 ± 16.9	56.9 ± 10.2	0.413	0.27	−0.36 to 0.90

**Table 4:** Serum BDNF.

	Pre (Mean ± SD)	Post (Mean ± SD)	*p*-Value	Cohen’s d	95% CI
**Serum BDNF (pg/mL)**	334 ± 193	408 ± 260	0.31	0.31	−0.30 to 0.95

## Data Availability

The original contributions presented in this study are included in the article. Further inquiries can be directed to the corresponding author(s).

## Abbreviations

The following abbreviations are used in this manuscript:

LM	Lion’s mane (*Hericium Erinaceus*)
BDNF	Brain-derived neurotrophic factor
NGF	Nerve growth factor
DCCS	Dimensional change card sort (test)
MMSE	Mini-Mental State Examination
ELISA	Enzyme-linked immunosorbent assay
BMI	Body mass index
SD	Standard deviation
IRB	Institutional review board
BRANY	Biomedical Research Alliance of New York
SPSS	Statistical Package for the Social Sciences (IBM SPSS)
NIH	National Institutes of Health (as in NIH Toolbox)
CI	Confidence interval
